# Cardiovascular alterations in rats with Parkinsonism induced by 6-OHDA and treated with Domperidone

**DOI:** 10.1038/s41598-019-45518-z

**Published:** 2019-06-20

**Authors:** Laís D. Rodrigues, Leandro F. Oliveira, Lucas Shinoda, Carla A. Scorza, Jean Faber, Henrique B. Ferraz, Luiz R. G. Britto, Fulvio A. Scorza

**Affiliations:** 1 Department of Neurology and Neurosurgery, UNIFESP/EPM, São Paulo, Brazil; 2 Laboratory of Cellular Neurobiology, Department of Physiology and Biophysics -University of São Paulo, São Paulo, Brazil

**Keywords:** Cardiology, Parkinson's disease, Parkinson's disease

## Abstract

After Alzheimer, Parkinson disease (PD) is the most frequently occurring progressive, degenerative neurological disease. It affects both sympathetic and parasympathetic nervous systems in a variable fashion. Cardiovascular symptoms are present in almost all stages of PD and narrower heart rate variability is the earliest sign. Administration of Levodopa to PD patients has proven to provide some degree of neurological protection. This drug, however, causes side effects including nausea and vomiting, lessened by the administration of domperidone. Autopsies in PD patients led some researchers to suggest the involvement of the ventricular arrhythmia induced by domperidone. The aim of the present study was to determine the impact of the adjusted human maximal dose of domperidone, on cardiological features of Wistar rats. domperidone was administered to both 6-hydroxydopamine Parkinsonism models and regular Wistar rats. Quantitative analysis of ranges of heart beat variation showed significant abnormal distribution in both groups receiving domperidone as compared with respective sham counterparts. However, qualitative analysis of Poincaré plots showed that 6-hydroxydopamine Parkinsonism models receiving domperidone had the narrowest full range of heart beat and the worst distribution heart beat ranges as compared with all study groups corroborating with previous suggestion that domperidone administration to PD patients is likely to play a role in sudden unexpected death in this group of patients.

## Introduction

Parkinson disease (PD) is the second most frequently occurring progressive degenerative neurological disease. It was first reported by James Parkinson in 1817 in his “An Essay on the shaking palsy”^1–3^. PD is characterized by the loss of dopaminergic neurons in the substantia *nigra pars compacta*, impairing the nigrostriatal dopaminergic pathway signaling, particularly with that going to the putamen^4,5^. In post-mortem examination, aggregate of α-synuclein protein (Lewy body) is found intracellularly^6,7^. Clinical manifestations in PD are tremor at rest, rigidity, bradykinesia, postural instability, freezing and autonomic dysfunctions culminating with depression and dementia^2,8^. The autonomic dysfunctions are variable and affect both sympathetic and parasympathetic nervous systems^9,10^ encompassing the cardiovascular, gastrointestinal, urogenital systems, sudomotor and thermal regulation, pupillary control and sleep and respiratory activities^11,12^. Cardiovascular symptoms are present in almost all stages of PD^3^ and heart rate variability seems to be a key feature becoming less variable before any motor symptom suggests PD^13^.

Parkinson patients most frequently die from pneumonia, cerebrovascular and cardiovascular disease, but there are risk factors for premature or sudden unexpected death in Parkinson Disease (SUDPAR) which include time length of motor PD signals, gender, motor severity, sleep disorders, concomitant cardiac and pulmonary disease, and increased number of treatment drugs^14,15^.

One drug in particular raises special concern. As soon as PD is diagnosed, the administration of Levodopa is normally initiated^16^. Although levodopa is quite effective in controlling motor symptoms there may be side effects, which include nausea, reflux and vomiting. In order to lessen these referred uncomfortable side effects, domperidone is prescribed^17^. Domperidone, a drug with primary peripheral nervous involvement, produces an antiemetic action by blocking the dopamine_2_-receptors (D2) in smooth muscles, thus reducing levodopa’s excitatory effects on the musculature of the gastrointestinal tract responsible for nausea, reflux and vomiting^17,18^. However, domperidone has been reported to cause ventricular arrhythmia by delaying the repolarization of the cardiac muscle producing a prolonged QT interval^17^.

The direct analysis of electrocardiographs is a time consuming task, but an alternative method adapted to computer processing can overcome this drawback. It is the analysis of heart rate variability (HRV), a tool of choice among biomedical researchers^19^ The effects of D2 antagonist on cardiac autonomic modulation have been previously suggested in healthy humans using HRV, through sympathetic nervous system stimulation^20^.

The aim of the present study was to determine the impact of the adjusted maximal dose of domperidone, normally prescribed for adult humans, on the heart rate variability of Wistar rats subject to 6-hydroxydopamine Parkinsonism model.

## Results

### Cylinder test

**Figure 1:**
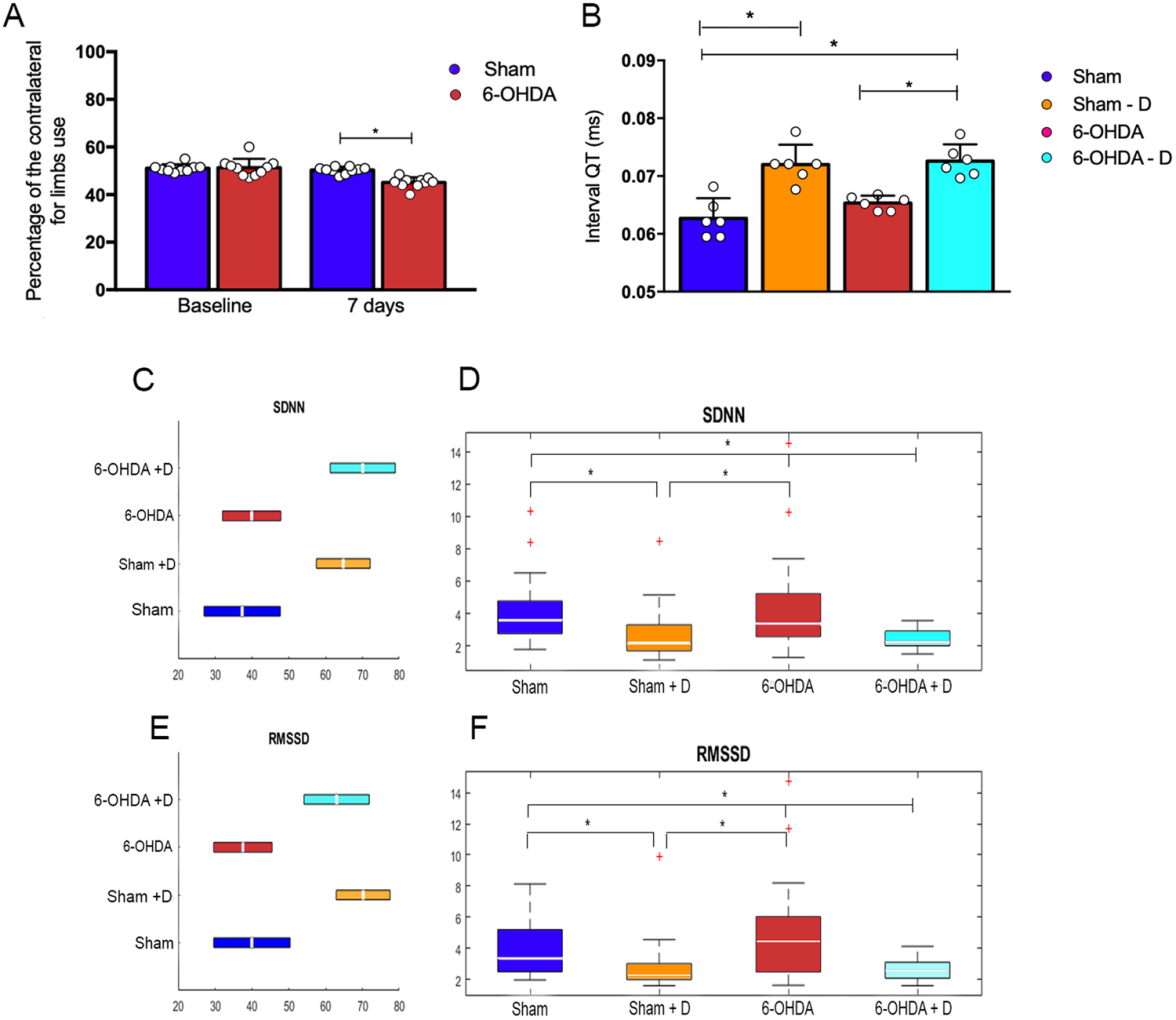
Statistical analysis of group measurements of SDNN, RMSSD and QT interval. (**A**) Cylinder test results showing percentage of contralateral forelimbs - staples indicate statistically significant difference between groups (**B**) Median lengths of QT interval (dots in color) indicate the distribution of individual values in the group – staples indicate statistically significant difference between groups (*p < 0.05). (**C**,**E**) show the ranges of variation (horizontal bars) of SDNN and RMSSD mean values obtained for each individual in a group. The configuration of the ranges within a group are consistent with the findings of Dunn & Sidàk ranking test, *i*.*e*., the range of variation of both SDNN and RMSSD mean values are not aligned when referring to groups showing statistically significant difference; (**D**,**F**) Box plot representation (median value and quartiles) of SDNN and RMSSD, respectively (rates with non-parametric distribution). These 2 parameters are used to characterize the distribution of intervals R-R by ranges and the discrepancy between two successive R-R intervals, respectively. Staples indicate statistically significant difference between group rates according to *post hoc* Dunn & Sidàk ranking test for *p < 0.05.

The administration of 6-OHDA was effective to cause brain injury to the right hemisphere as ascertained by predominant use of the right forepaw, which was determined by the statistically significant number of touches of this limb on the cylinder wall as compared with the left one, 7 days after the injection of this drug (p < 0.0001 and *t* = 6.276). The injection of saline (sham group) produced no changes in expected normal use of forelimbs (Fig. 1A).

### Variability analysis

Standard deviation (SD) of R-R intervals distribution (SDNN) and square root of mean squared differences of successive R-R intervals (RMSSD) were both significantly different when comparing Sham + D with Sham and with 6-OHDA, and when comparing 6-OHDA + D with Sham and with 6-OHDA (H = 21.70 and p < 0.001 for SDNN; H = 23.43 and p < 0.001 for RMSSD) (Fig. 1C–F, Tables 1 and 2).

**Table 1:** Group statistics. Median values of SDNN, RMSSD, SD1 and SD2 obtained within groups (non-parametric distribution) Sham, Sham + Domperidone, 6-OHDA and 6-OHDA + Domperidone along with respective interquartile ranges (iqr).

	Sham		Sham + D		6-OHDA		6-OHDA + D	
	Median	iqr	Median	iqr	Median	iqr	Median	iqr
*SDNN*	3.622	2.008	2.169	1.596	3.386	2.640	2.229	0.909
*RMSSD*	3.356	2.698	2.236	1.025	4.457	3.532	2.584	1.031
*SD1*	2.396	1.928	1.597	0.734	3.186	2.520	1.842	0.738
*SD2*	4.374	2.943	2.471	2.083	3.386	3.000	2.683	1.855

**Table 2:** Group median rates and standard errors obtained in Kruskal-Wallis rank analysis.

	Sham		Sham + D		6-OHDA		6-OHDA + D		H	p-value
	Median	se	Median	se	Median	se	Median	se		
*SDNN*	70.08	6.39	39.95	5.72	64.81	5.22	37.36	7.38	21.70	<0.001
*RMSSD*	63.00	6.39	37.57	5.72	70.19	5.22	40.00	7.38	23.43	<0.001
*SD1*	63.00	6.39	37.55	5.72	70.22	5.22	39.97	7.38	23.50	<0.001
*SD2*	70.42	6.39	42.38	5.72	60.50	5.22	41.47	7.38	15.12	<0.001

**Figure 2:**
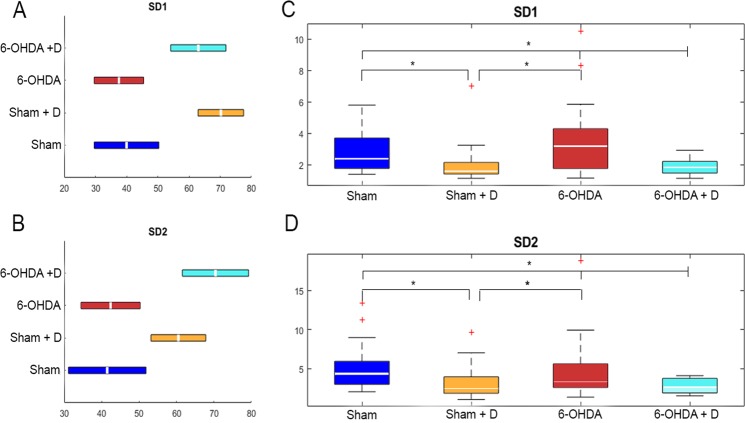
Statistical analysis of group measurements of SD1 and SD2. (**A**,**C**) Box plot representation (median value and quartiles) of SD1 and SD2 (axis of the ellipsis fit on Poincaré scatter plot configuration), respectively (rates with non-parametric distribution). Staples indicate statistically significant difference between group rates according to *post hoc* Dunn & Sidàk ranking test for *p < 0.05. (**C**,**D**) Show the ranges of variation (horizontal bars) of SD1 and SD2 mean values obtained for each individual in a group. The configuration of the ranges within a group are consistent with the findings of Dunn & Sidàk ranking test, *i*.*e*., the range of variation of both SD1 and SD2 mean values are not aligned when referring to groups showing statistically significant difference

Standard deviations of Poincaré dots from minor and major ellipsis axes (SD1 and SD2) were both significantly different when comparing Sham + D with Sham and with 6-OHDA, and when comparing 6-OHDA + D with Sham and with 6-OHDA (H = 23.50 and p < 0.001 for SD1; H = 15.12 and p < 0.002 for SD2) (Fig. 2, Tables 1 and 2).

### QT interval analysis

The length of QT interval was significantly increased in 6-OHDA + D animals as compared with Sham and 6-OHDA (H = 17.83 and p = 0.0005). No other statistically significant difference among groups was found (Fig. 1B).

### Immunohistochemistry

**Figure 3:**
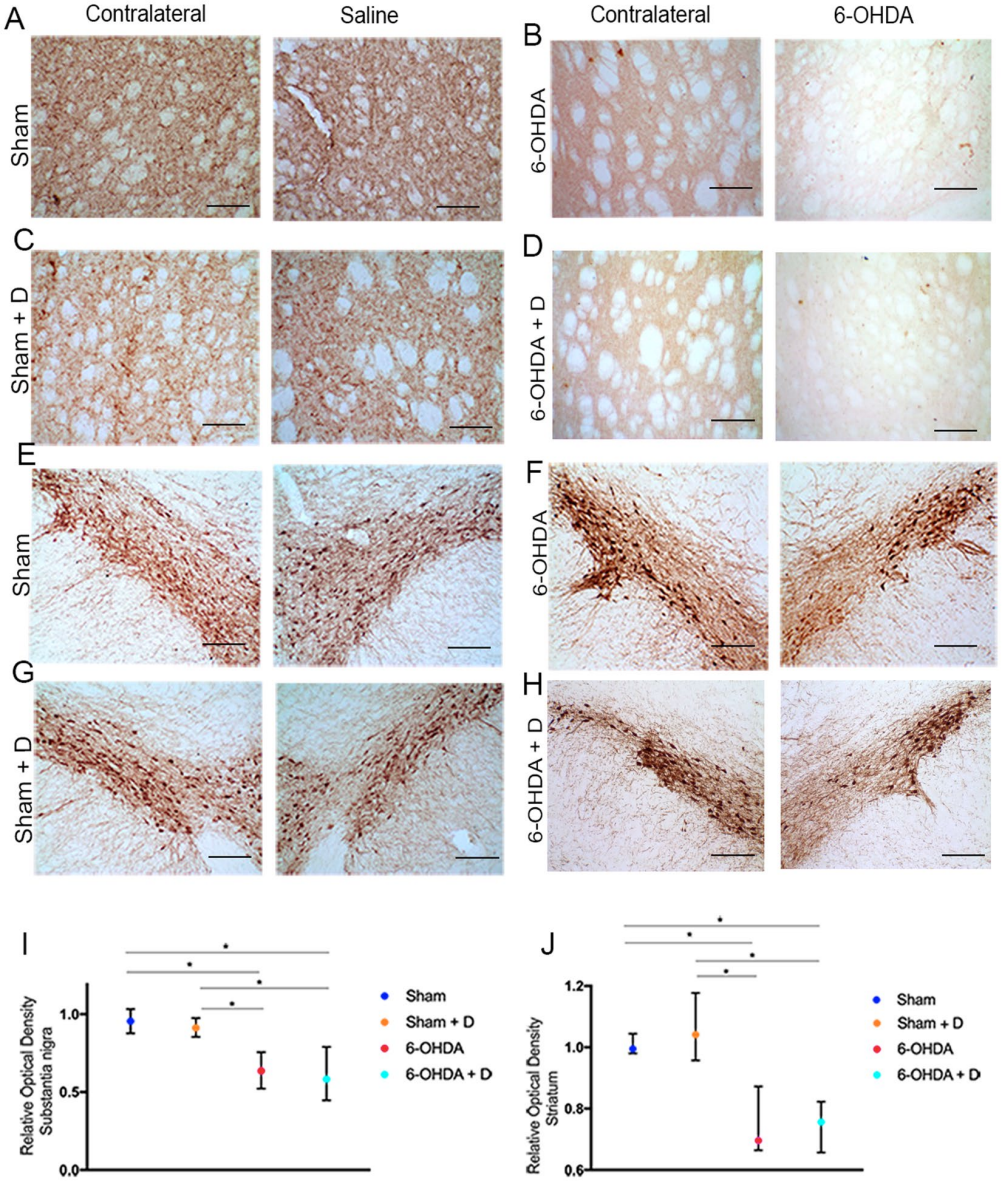
Immunohistochemical staining and analysis. (**A**–**D**) Caudate-putamen complex staining (bar in the right-hand low corner indicate 200 µm); (**E**–**H**) *Substantia nigra pars compacta* staining (bar in the right-hand low corner indicate 200 µm); (**I**,**J**) Statistical analysis – staples indicate statistically significant difference between groups (*p < 0.05).

Groups 6-OHDA and 6-OHDA + D were significantly different from groups Sham and Sham + D regarding neuronal loss as assessed by optical analysis of staining intensity. Striatum (H = 44.12 and p < 0.001) *Substantia nigra* (H = 49.18 and p < 0.001) (Fig. 3).

## Discussion

PD patients die from different causes including aspiration pneumonia, dementia, old age, cancer, cardiovascular disease and sudden unexpected death^21–23^. Several studies have shown that changes in cardiac function may be present in PD patients^12,24^. Scorza *et al*. emphasize the importance of a regular cardiological screening including electrocardiogram, echocardiogram and Holter monitoring for palliative care of patients diagnosed with PD as the findings of these screening, besides providing a better guiding to a multidisciplinary approach in treating PD, could also shed some light on whether this condition may also involve sudden unexpected death (SUDPAR – Sudden Unexpected Death In Parkinson Disease)^3^.

The Parkinson disease animal model used in the current study was Parkinsonism induced by 6-OHDA, as the toxicity of the Parkinson-like lesion inducing drug is relatively selective for dopaminergic neurons^2,25^. 6-OHDA was injected in the striatum, because according to previous studies^26,27^, Parkinsonism is characterized seven days after injections. In the present study, motor signs appeared, as shown in cylinder test, one week after drug injection. (Fig. 1A).

Immunohistochemistry assay soon after study endpoint (day 15) showed Parkinson-like degeneration in the Caudate and Putamen (CPu), and *Substantia Nigra pars compacta* (SNpc) in groups 6-OHDA and 6-OHDA-D as ascertained by comparing these regions with the non-affected contralateral corresponding structures (Fig. 3A–J)

PD is known to decrease patient’s heart rate variability (HRV)^28,29^. Several studies on cardiologic conditions reports an association between reduced HRV with sudden unexpected death^30^. Heart rate variability is the variability of the distance between two successive peaks R of the complex QRS measured on the electrocardiograph, and is commonly referred to as R-R interval^31^. The interaction of sympathetic and parasympathetic activities, the latter sometimes mentioned as vagal activity, during heart functioning reflects on this variability^32^.

A number of indices are used to assess the cardiac function, e.g. in patients with epilepsy the square root of the squared mean differences between successive R-R intervals (RMSSD) was associated with the risk of sudden unexpected death^33^.

Another index is the standard deviation of the distribution of R-R intervals (SDNN) along the full range of heart beat frequency. In the present study, the treatment with domperidone to Wistar rats with Parkinson-like lesion and sham animals to obtain groups 6-OHDA + D and Sham + D, respectively, significantly reduced the SDNN and RMSSD rates in these groups as compared with rates determined for their respective controls, 6-OHDA and sham (Fig. 1D,F). Studies including Parkinson patients in treatment, when domperidone is commonly prescribed to suppress reflux caused by Parkinson medication^17^, reported a smaller SDNN and RMSSD as compared with controls^34–37^. Furthermore, Maheshwari *et al*., studying atherosclerotic coronary heart disease in general population and using SDNN and RMSSD as HRV coefficients found an expressive correlation between sudden cardiac death (SCD) and lower ranges of SDNN and RMSSD^30^.

An important ECG feature is the interval QT, which is measured on the electrocardiograph from the beginning of Q until the end of T waves^38^. In cardiac arrhythmia, ventricular tachycardia and some cases of SCD, interval QT showed prolonged^39^. Bruera *et al*.^40^ observed an increased QT interval in a 57-year-old female patient with stage III ovarian cancerous tumor, who had been administered 60 mg of domperidone intravenously, experienced ventricular tachycardia and fibrillation. In similar fashion, the present Sham + D and 6-OHDA + D animals exhibited prolonged QT intervals as compared with sham and 6-OHDA ones. In the attempt to elucidate the prolongation of interval QT caused by domperidone, Drolet *et al*. performing *in vitro* experimentations suggested that this drug can disturb the repolarization of the cardiac muscle by interfering in the expression/translation of potassium channels^41^. Despite the great majority of studies in patients reporting domperidone as prolonging QT interval, Biewenga *et al*.^42^ raises controversy reporting domperidone not to impact on QT interval in health subjects. Once again, the interaction of domperidone with other therapy drugs have been reported to worsen QT prolongation^43^. Furthermore, domperidone, still used as the first choice therapy for gastrointestinal symptoms in PD, has been implicated as cardiotoxic in doses exceeding 30 mg/day^17^.

Two different studies by Renoux *et* *al*. reported that PD patients taking domperidone had higher risk of cardiac conditions as compared with those not taking it. Their 2012 study suggested that the group of 19% of patients affected with cardiac failure would be in greater risk in case they had been given domperidone^44^. A later study by the same authors showed that those taking this drug had a 22% increased risk for ventricular tachyarrhythmia and SCD as compared with controls^45^.

**Figure 4:**
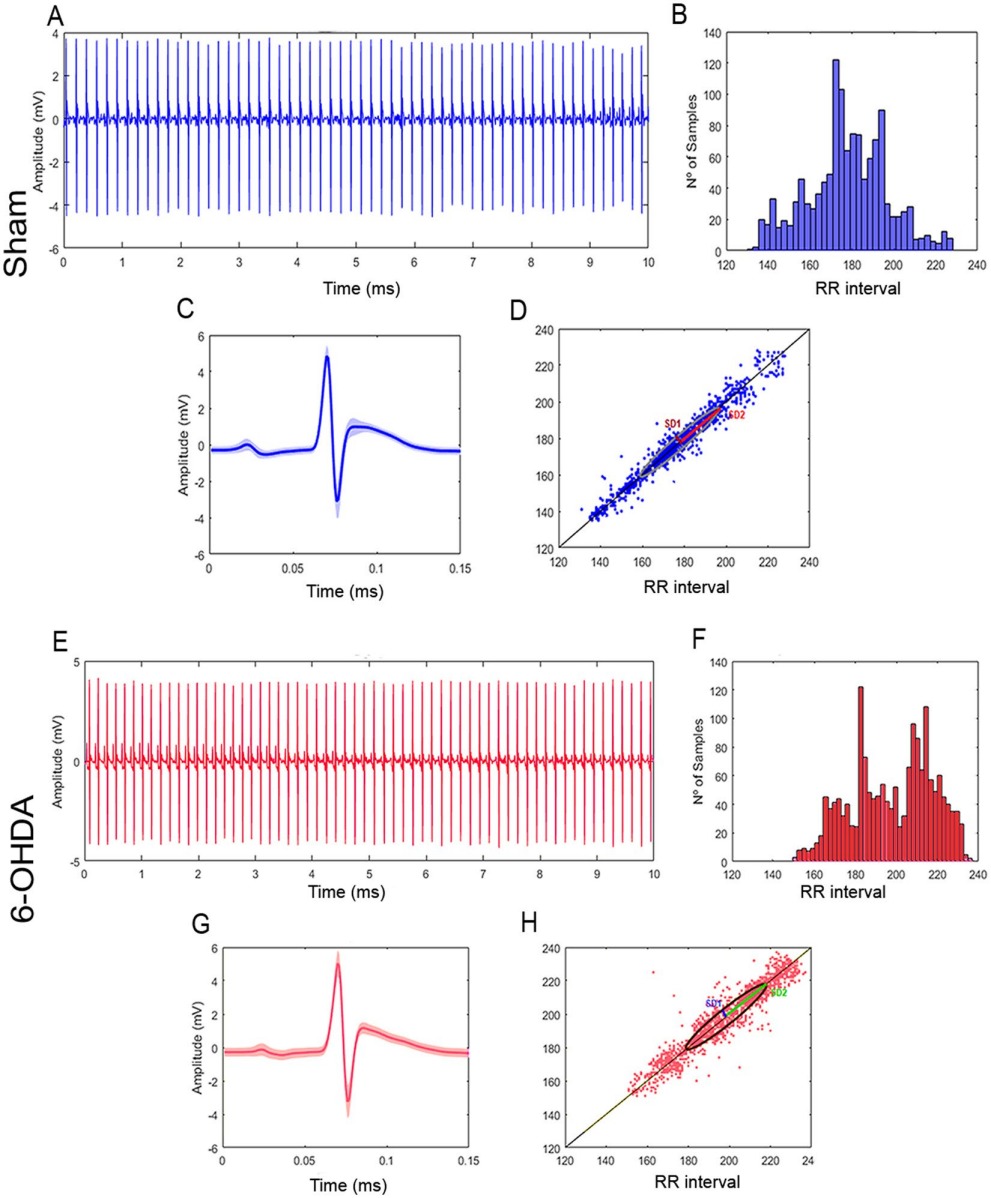
Group characterization. Sham (**A**) Electrocardiograph with R-R peaks evidenced (blue); (**B**) Distribution of R-R intervals by length range; (**C**) Superimposition of P-Q-R-S-T complexes to determine variation in shape; (**D**) Poincaré scatter plot displaying the shape the ellipsis fitted. 6-OHDA (**E**) Electrocardiograph with R-R peaks evidenced (blue); (**F**) Distribution of R-R intervals by length range; (**G**) Superimposition of P-Q-R-S-T complexes to determine variation in shape; (**H**) Poincaré scatter plot displaying the shape the ellipsis fitted. 6-OHDA.

Despite indices such as SDNN are commonly used to characterize morbidity and heath findings, sometimes they cannot differ between these two conditions, *e*.*g*.in the present study there was no statistically significant difference between SDNNs calculated for sham and 6-OHDA groups, although their histograms of distribution of R-R intervals (or alternatively frequencies) were clearly different. Whereas sham group showed frequency ranges with higher occurrence rates in central position (180 ms) (Fig. 4B), 6-OHDA animals had frequency ranges with higher occurrence rates clustered in three different positions between the minimum and maximum rates of R-R interval (placed at around 175, 195 and 215 ms) (Fig. 4F). It is reasonable to assume that a change in the distribution of occurrences of R-R intervals for healthy individuals will probably be associated with a not-so-heathy condition.

The current study found that SD1 indices were not statistically different when determined for groups sham and 6-OHDA, nor SD2 (Fig. 2C,D). The same was determined when comparing sham + D and 6-OHDA + D animals (Fig. 2C,D). However, SD1 and SD2 determinations for sham + D and 6-OHDA + D were reduced with statistical significance as compared with those determined for sham and 6-OHDA (Fig. 2C,D). The present findings are similar to those reported by Estanol *et al*., who obtained decreased rates of SD1 and SD2 for in-treatment Parkinson patients as compared with healthy controls. These authors correlated their findings with cardiosympathetic and cardiovagal damage^35^.

An attempt to find another numerical analysis of the HRV data obtained from the four study groups was made by applying the Kolmogorov-Smirnov test to compare the four distributions of RR intervals 2 at a time. Unfortunately, the only information obtained from the statistics software used was that the probability of any distribution being similar to any other was less 0.001 (Table 3).

**Table 3:** Kolmogorov-Smirnov test for distributions. The comparison of the RR interval distributions obtained for the four study groups two at a time aplying the Kolmogorov-Smirnov test resulted that the probability of any distribution being similar to any other was less 0.001.

		Sham	Sham + D	6-OHDA	6-OHDA + D
Histogram	Sham		p < 0.001***	p < 0.001***	p < 0.001***
	Sham + D	p < 0.001***		p < 0.001***	p < 0.001***
	6-OHDA	p < 0.001***	p < 0.001***		p < 0.001***
	6-OHDA + D	p < 0.001***	p < 0.001***	p < 0.001***	

Similarly to the current contradicting findings involving the quantitative measures SDNN and RMSSD and the qualitative analysis of respective distribution of R-R intervals, current SD1 and SD2 indices did not show statistically significant difference when comparing groups sham with 6-OHDA, and sham + D with 6-OHDA + D. However, the visual (qualitative) examination of Poincaré scatter plots^46^ (Psp) themselves show clear differences in heart beat behaviors when comparing these groups as referred. Differently from RMSSD, which is a measure of overall variance of R-R interval along minimum-to-maximum rates and therefore cannot describe regional variations^47^, or from Poincaré SD1 and SD2 indices, which are standard deviations also referenced to the mean rates and, once again, cannot describe regional variability along the minimum-to-maximum interval^48^, the Poincaré plot itself provides a better, though visual, idea of both global and regional behavior of HRV^49^.

**Figure 5:**
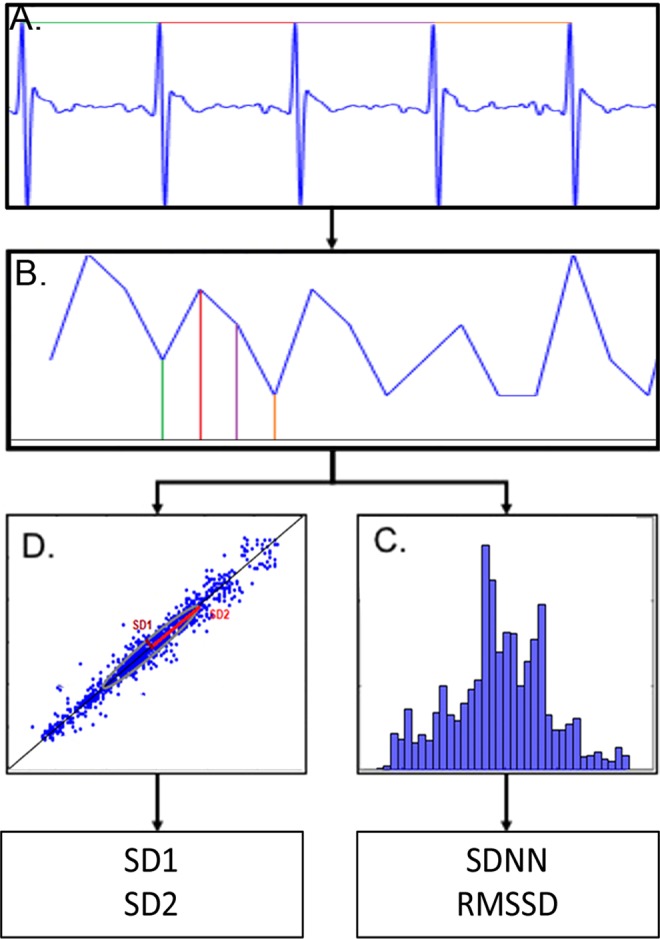
Flow chart of data analysis. (**A**) ECG signal acquisition; (**B**) Tachogram depicting the determination of R-R intervals over time; (**C**) Assembling of R-R interval distribution by length range and determination of SDNN and RMSSD indices; (**D**) Cartesian plotting of consecutive R-R intervals (Poincaré scatter plot) and determination of SD1 and SD2 indices.

Poincaré scatter plot is the charting of dots on the cartesian plane using as X and Y coordinates the lengths of two consecutive R-R intervals (referred simply as RR from this point on) RR_n_ and RR_n+1_, respectively^46^. The discrepancy between or difference in their lengths (RR_n+1_-RR_n_) is shown as the distance of their corresponding cartesian dot from the 45-degree-slopped straight line that crosses the intersection between X and Y cartesian axes, taken on a parallel to Y axis (Fig. 5D)^50^. The farther away from the slopped line the dot is, the greater the discrepancy between a RR and its consecutive element is. Small RR discrepancies are expected in healthy individuals. It is also expected the dots to lie very close to the slopped line, either above or below it, tending to produce a continuous solid marked line. Heavier concentration of dots indicating ‘preferable’ range of heart beating rate is distinguished by a deeper tinge on a certain stretch of this line^51^.

The Poincaré plot obtained for a healthy Wistar rats (group sham) is the reference configuration for the analysis of the cardiac functioning in the other groups. As compared with group sham that shows a full-length heart rate range running from 140 to 230 ms (Fig. 4D), Parkinson animals exhibit a bradycardic pattern with heart rate range of 155 to 240 ms, bearing in mind that slower heart beat corresponds to longer RR interval (Fig. 4H). In addition, the dots are slightly more distant from the 45-degree straight line, clearly more scattered and displaying four stretches with clustering tendency (at approximately 170, 190, 215 and 230 ms) (Fig. 4H). Physiologically, scattering and deviation of the dots from the 45-degree line can be associated with an increase in the number of heart beat irregularity that together with the four different points showing tendency of clustering leads to the assumption that the regulatory system of the cardiac function is someway failing.

**Figure 6:**
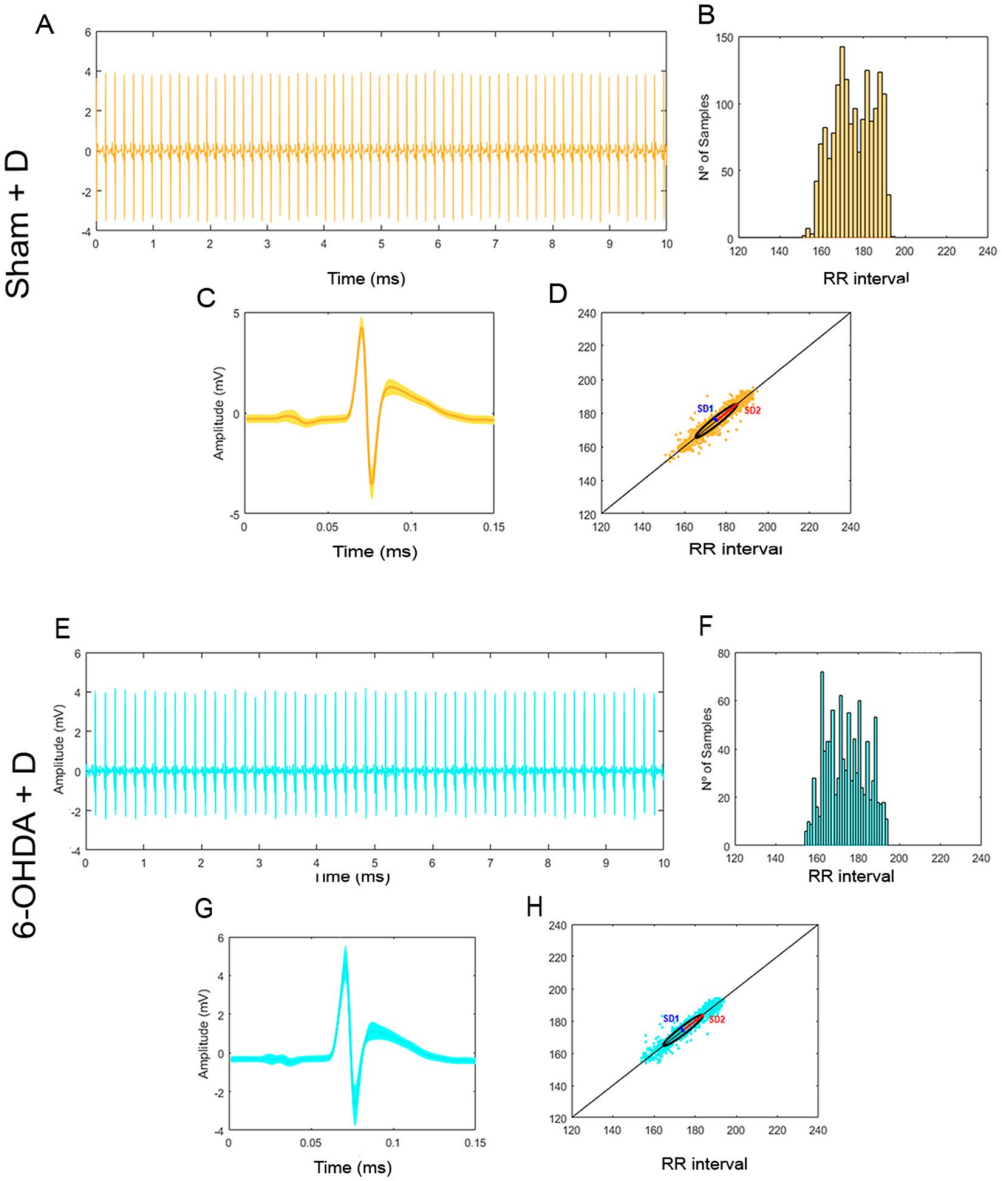
Group characterization. Sham + D. (**A**) Electrocardiograph with R-R peaks evidenced (blue); (**B**) Distribution of R-R intervals by length range; (**C**) superimposition of P-Q-R-S-T complexes to determine variation in shape; (**D**) Poincaré scatter plot displaying the shape the ellipsis fitted. 6-OHDA = D (**E**) Electrocardiograph with R-R peaks evidenced (blue); (**F**) Distribution of R-R intervals by length range; (**G**) superimposition of P-Q-R-S-T complexes to determine variation in shape; (**H**) Poincaré scatter plot displaying the shape the ellipsis fitted. 6-OHDA.

The administration of domperidone to 6-OHDA animals resulted in a Poincaré scatter plot with significant reduction in the full-length range of heart rate variation of around 43%, and in an increase in the deviation of the dots from the 45-degree line of about 57%. In this group the combination of both features findings seems to indicate a significant impairment of the mechanisms that regulate the cardiac function (Fig. 6).

Our findings suggest that traditional quantitative measurements of heart rate variability (HRV) – SDNN, RMSSD and Poincaré indices SD1 and SD2 – may not be enough to describe the severity of the impairment in the functioning of the cardiac muscle. On the other hand, when associated with the visual examination of heart beat patterns as provided by the Poincaré scatter plot, a clearer picture of the cardiac condition can be revealed, as is the case of the administration of domperidone to animal model of parkinsonism. We acknowledge that the animal model used in the present research work has a pattern of HVR intrinsic to neurological injury caused by the particular administration of 6-OHDA, and that relevant studies on HVR patterns displayed by PD patients over the progression of the disease should be performed. Simeonova *et al*. found a 2-fold death risk in PD patients taking domperidone as compared with those PD patients not taking this drug^52^. Thus, performing screenings of PD patient’s HRV over the course of the disease could greatly improve the management of the disease and perhaps provide further clues to whether or not the administration of domperidone to Parkinson’s patients is associated with sudden unexpected death. Once acquiring the digital ECG data, free software tools can fast and easily provide a Poincaré scatter plot, an invaluable aid to detail the cardiac condition in Parkinson’s disease.

## Methods

### Animals

A total of 36 male, adult Wistar rats weighing 230 g to 300 g from the experimental animal center of the Federal University of São Paulo –UNIFESP, housed (four per cage) at temperature, of 21° ± 2 °C, and light and dark cycle of 12 hours with free access to food and water were used in this study.

All animal handling and experimental procedures complied with the guidelines for animal care and use of laboratory animal and received the approval from the Board for Ethics in the Use of Animal (CEUA – Comissão de Ética no uso de animais) an institutional ethics committee of the Federal University of São Paulo (UNIFESP) under number 6463110417.

### Study design

Animals were divided into four groups 6-OHDA, 6-OHDA-D, Sham, Sham-D. Parkinson-like injury was induced in groups 6-OHDA, 6-OHDA-D by injecting 6-hydroxydopamine (6-OHDA) in the striatum, whereas Sham and Sham D animals were injected saline with ascorbic acid (day 0). Animals in groups 6-OHDA-D and Sham-D were treated with domperidone (80 mg/kg) via gavage for 7 days (from day 8 through day 14). A daily 60-minute electrocardiographic (ECG) recording was performed from day 8 through day 13 at the same time for animal familiarization and use the recording on day 14 for analysis. Perfusion with phosphate buffer saline (PBS) and paraformaldehyde at 4% for immunohistochemistry of brain tissue was performed at study endpoint (day 14).

### 6-OHDA injection and ECG electrode implantation

Pre-operative procedures included the administration of Xilazine (10 mg/kg-Anesadan™), and Ketamine (100 mg/kg Dopalen™).

Having installed the stereotaxic device (EFF 331- Insight™), incision, boring and injection of 0,5 µL of 6-OHDA cloridate (Sigma©) prepared in saline solution of ascorbic acid at 0,3% at concentration of 6 µg/µL were performed at coordinates AP: 0.0, LL:-2.7, DV:-4.5 and AP:0.5, LL:-3.2, DV-4^53,54^ (right hemisphere) in 6-OHDA and 6-OHDA-D animals^26,27^. The same procedures were performed in Sham and Sham-D animals excepting that only saline solution of ascorbic acid at 0.3% was injected.

**Figure 7:**
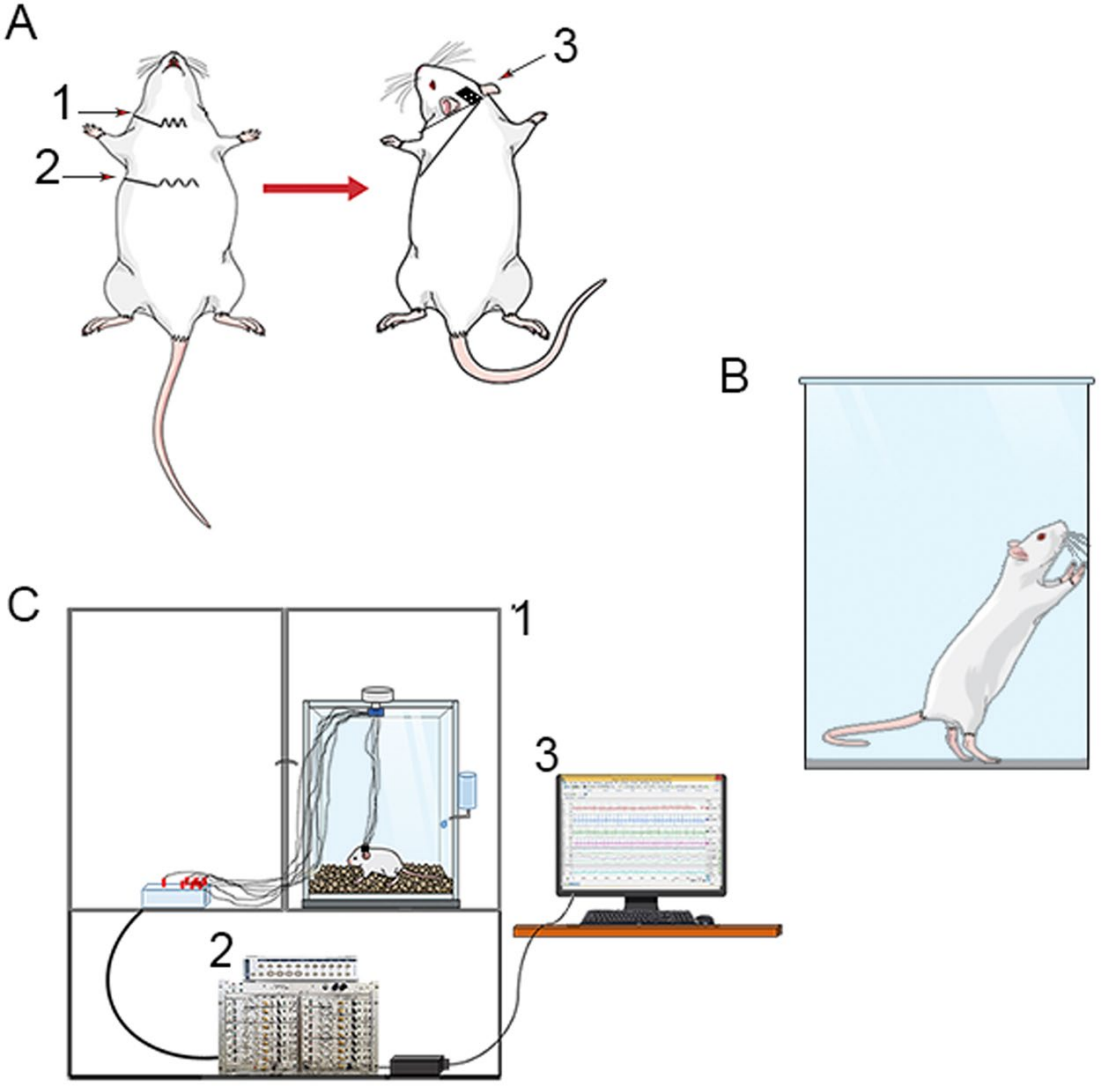
ECG recording – (**A**) Subcutaneous implantation of electrodes in the sternocleidomastoid (1), xiphoid process (2) and leads for signal recording. (**B**) Faraday cage (1); amplifier of signals (below) and digitizer (3) and signal monitoring. Figure was created in the Mind the Graph platform http://www.mindthegraph.com under Creative Commons License CC community as “attribution share-alike 4.0 licensing”: https://creativecommons.org/licenses/by/4.0/.

Following, a micro connector for ECG leads was attached to the animal’s skull with dental base acrylic resin. Two ECG leads (nylon coated steel wire) from the connector were guided subcutaneously to be fixed one to the xiphoid process and the other to sternocleidomastoid muscle (Fig. 7A,C) (adapted: Sgoifo *et al*. 1996)

After operative procedures, Tramadol HCl (5 mg/kg) and meloxicam (2 mg/kg) were administered subcutaneously, and animals were housed and observed.

### Cylinder test

The cylinder behavior test was used to ascertain favorite use of one of the forelimbs after unilateral injury to right motor nuclei in the central nervous system caused by the injection of 6-OHDA. The protocol was performed before experimental day 0 and 7 days after the injection of 6-OHDA. The animals were left into a transparent acrylic cylinder 20 cm in diameter and 30 cm in height and their exploratory behaviors was observed for 5 minutes in order to determine the number of touches on the cylinder wall with single right and left forepaw during their standing on rear limbs. Only animals with statistically significant preference for the right forepaw were counted among animals with Parkinsonism and included in group 6-OHDA^27^ (Fig. 7B).

### Domperidone/saline treatment

Domperidone (80 mg/kg – Medley© adjusted for rats^55^) or saline was administered by gavage to 6-OHDA-D and Sham-D animals and 6-OHDA and Sham animals, respectively from day 7 to day 14.

### ECG data acquisition

Animals were placed in individual boxes for ECG recording. Signals were amplified, digitized and recorded using PowerLab 8/35 (Adinstruments, Australia). Visualization and pre-filtering were obtained by using LabChart V8 software (ADinstruments, Australia). Recording was performed at sampling rate of 1000 Hz during 60 minutes in experimental day 14. MATLAB ™ software and a computer with 8GB RAM, Intel® Core ™ i7-6700 processor, 3.4 GHz were used to process ECG signals.

### Quantitative and qualitative description of heart rate variability

For each animal six stretches of the ECG recorded were selected and the time elapsed (in milliseconds) elapsed between two consecutive heart beats along each of these stretches were determined as the time elapsed between two consecutive peaks R (as defined electrocardiographically in the complex QRS) from this point on referred to as R-R interval. All R-R intervals determined in each animal group were distributed in ranges and plotted for visualization. For each stretch in every animal group (a total of 24 stretches in each group), the mean R-R interval and the standard deviation of R-R intervals (SDNN) were determined. Additionally, a measure of variance defined as the square root of the squared mean difference between successive R-R intervals (RMSSD) was also determined (Fig. 5).

Similarly, Poincaré scatter plots for each animal group were generated for visualization and qualitative analysis. For each stretch in every group and SD1 and SD2 (standard deviations of dots plotted from minor and major ellipse axes, respectively) were determined for quantitative analysis.

### QT interval analysis

In order to verify the reported prolongation of QT interval in patients using domperidone, the length of QT interval for all groups was obtained from ECG data acquired as standard data provided by the software application LabChart V8 used.

### Immunohistochemistry

At study endpoint (day 14), 4 animals from each group received a lethal dose of sodium thiopental (80 mg/kg – Thiopentax) and were perfused transcardially from the left ventricle, first with 100 ml PBS (0,1 M – pH7.4), and after with 150 ml paraformaldehyde at 4% in PBS. Their brains were then extracted, post fixed in paraformaldehyde at 4% for 24 hours and kept in sacarose PBS solution at 30%, for 72 hours. Sections of 40 µm were obtained in cryostat microtome (HE 505E - Microm). Sections were washed in PBS, and treated with hydrogen peroxide PBS solution at 1% for 30 minutes, followed by Triton-X at 0.3% for 30 minutes, and kept in albumin solution at 10% for 3 hours followed by overnight incubation at 4 °C with primary antibody Tyrosine Hydroxylase in 2% albumin solution, (1:1500 – Abcam – AB112), overnight. Subsequently, sections were incubated for 2 hours with a secondary antibody (biotinylated anti-rabbit IgG – Abcam - AB6721 - 1:200) at room temperature, next the slices were subjected to the diaminobenzidine tetrahydrochloride (DAB) reaction, diluted in Tris-HCL (pH- 7.6).

### Quantification of neuronal injury

Neuronal injury to the Substantia Nigra pars compacta (SNc) and Caudate Putamen Complex (CPu) caused by the injection of 6-OHDA in the right brain hemisphere was determined by comparing the immunohistochemical staining of the functional neuronal cells in the healthy and injured SNc and CPu portions, in the left and right hemisphere, respectively.

The intensity of immune-labeling was determined by optical density measured on digital images of 40μm brain sections, treated as above mentioned, acquired from a Nikon E1000 microscope (Melville, NY, USA).

A total of 5 sections per brain, taken 150μm apart in order to cover the most possible of the aimed structures in both hemispheres, were used. In the particular case of the CPu, the optical density of the coloration was measured in 3 areas of 0.0050μm that did not include any fiber bundles.

### Statistical analysis

All sets of data in this study excepting those concerning QT interval and cylinder test did not have normal distribution as determined by the Shapiro-Wilk test.

Non-parametric data was analyzed for statistical difference among groups using Kruskal-Wallis test with *post hoc* test of Dunn & Sidàk.

Student’s t-test was used to ascertain statistical difference between two sets of data with normal distribution.

RR interval distributions were compared for likeness by applying the Kolmogorov-Sminorv test for distributions.

Comparisons between groups concerning standard deviation of RR intervals (SDNN), variance as RMSSD, standard deviations SD1 and SD2 in Poincaré plotting used 24 determinations of the variable under analysis in each group (4 animals having each 6 stretches of ECG recording sampled).

Level of statistical significance was set to 0.05.
